# Role of combination immunotherapy in restoring brain synergistic functional connectivity in patients with systemic lupus erythematosus without overt neuropsychiatric manifestations

**DOI:** 10.1136/lupus-2025-001771

**Published:** 2025-10-29

**Authors:** Yifan Yang, Cailin Liu, Shuang Liu, Peng Ding, Ru Bai, Gengyi Chen, Shu Li, Xiaopeng Song, Yuqi Cheng, Jian Xu

**Affiliations:** 1 Department of Rheumatology and Immunology, First Affiliated Hospital of Kunming Medical University, Kunming, Yunnan, China; 2 Department of Rheumatology and Immunology, The Second Affiliated Hospital of Nanchang University, Nanchang, Jiangxi, China; 3 Faculty of Information Engineering and Automation, Kunming University of Science and Technology, Kunming, China; 4 Brain Cognition and Brain-Computer Intelligence Integration Group, Kunming University of Science and Technology, Kunming, China; 5 Faculty of Medicine, Tianjin University Medical School, Tianjin, China; 6 Affiliated Mental Health Center, Zhejiang University School of Medicine, Hangzhou, China

**Keywords:** Systemic Lupus Erythematosus, Glucocorticoids, Anti-Inflammatory Agents, Non-Steroidal

## Abstract

**Objective:**

To determine whether subclinical brain dysfunction in SLE can be detected by disrupted interhemispheric connectivity and assess its modulation by immunosuppressive regimens.

**Methods:**

234 subjects (140 patients with SLE and 94 healthy controls (HCs)) were included. Through stratified analysis, patients with SLE were divided into treatment-naïve group (n=22), glucocorticoid monotherapy group (GC group, n=30) and GC combined with cyclophosphamide (CTX) and/or hydroxychloroquine (HCQ) treatment group (n=50) to assess the differences in voxel-mirrored homotopic connectivity (VMHC) between groups.

**Results:**

SLE group showed lower VMHC than the HC group in bilateral superior temporal gyrus, medial superior frontal gyrus, calcarine fissure and surrounding cortex and middle occipital cortices (Gaussian random field corrected: voxel p<0.005, cluster p<0.01). The VMHC in the bilateral superior temporal gyrus (r_s_=−0.250, p=0.024) and medial superior frontal gyrus (r_s_=−0.246, p=0.026) was negatively correlated with the depression score, while the VMHC in the medial superior frontal gyrus was negatively correlated with the anxiety score (r_s_=−0.239, p=0.031). Three SLE subgroups and HCs had different VMHC in the postcentral/precentral gyrus (*F*=8.942) and anterior cingulate/paracingulate gyrus (*F*=9.868). Post hoc analysis found that compared with the HC group, VMHC in the treatment-naïve group was decreased in the bilateral posterior central gyrus (*t*=−2.953), while in the GC monotherapy group, it decreased in the posterior central gyrus (*t*=−2.999) and anterior cingulate/paracingulate gyrus (*t*=−2.999). Compared with GC combined with CTX and/or HCQ group, VMHC in GC monotherapy group was decreased in the postcentral gyrus (*t*=−2.999).

**Conclusion:**

Even without overt neuropsychiatric symptoms, patients with SLE exhibit impaired interhemispheric functional synergy that is partially reversed by combination immunosuppression, suggesting an early targetable brain pathway.

**WHAT IS ALREADY KNOWN ON THIS TOPIC**

**WHAT THIS STUDY ADDS**

- In patients with SLE, voxel-mirrored homotopic connectivity (VMHC; a measure of brain hemispheric coordination) decreased in language, emotion regulation and visual integration regions, correlating with depression/anxiety scores. Glucocorticoid (GC) treatment alone partially restored VMHC, while combining GC with hydroxychloroquine or cyclophosphamide restored it to healthy control levels. This provides the first imaging evidence that combined immunosuppressive therapy can reverse SLE-related subclinical brain network damage.

**HOW THIS STUDY MIGHT AFFECT RESEARCH, PRACTICE OR POLICY**

- VMHC serves as an objective, quantifiable biomarker for early detection of central nervous system involvement in SLE and for assessing therapeutic efficacy. This facilitates shifting treatment focus from symptom management to prevention. Supporting data warrant prospective trials to assess whether early initiation of hydroxychloroquine/cyclophosphamide regimens can prevent progression to significant neuropsychiatric disorders.

## Introduction

 SLE is a chronic autoimmune disease characterised by the presence of multiple pathogenic autoantibodies in the serum that can affect multiple organ systems.^1^ Neuropsychiatric SLE (NPSLE) is a common and serious complication of SLE, which can manifest as a variety of central and peripheral nerve symptoms such as headache, epilepsy, cognitive dysfunction and autonomic neuropathy, which are significantly associated with cognitive decline, disability and mortality.^2 3^ According to the literature, patients with SLE who have not yet developed major neuropsychiatric symptoms and have no obvious imaging abnormalities in conventional brain magnetic resonance are called patients with SLE without overt neuropsychiatric manifestations.^4 5^ Although numerous studies have confirmed that patients with NPSLE have abnormalities in the brain functional connectivity network,^6 7^ it remains unresolved whether subclinical damage to the central nervous system (CNS) has developed in such patients with non-major NPSLE. The answer to this question will determine the window period and treatment strategy for early intervention.

The existing clinical assessment system faces a double dilemma: traditional neuropsychological scales, such as Mini-Mental State Examination (MMSE) and Hamilton Depression Scale (HAMD), are not sensitive enough for subtle brain function impairment, while conventional structural MRI often shows ‘no abnormality’, resulting in about 30% of patients with SLE without overt neuropsychiatric manifestations being misjudged to have ‘normal brain function’. More critically, although glucocorticoid (GC) combined with immunosuppressants such as hydroxychloroquine (HCQ) and cyclophosphamide (CTX) is the routine treatment option for SLE, their protective effects on brain function remain controversial: some studies suggest that GC improves white matter integrity by inhibiting neuroinflammation, while others suggest that long-term high-dose GC may exacerbate hippocampal atrophy.^8^ The root of this controversy is the lack of two key pieces of evidence—biomarkers that objectively quantify brain network damage and direct evidence of the neuroprotective efficacy of drugs based on imaging assessments.

Based on this, this study focuses on two major scientific questions: Do patients with SLE without overt neuropsychiatric symptoms already have a synergistic impairment of transhemispheric function? How do different drug regimens (especially GC in combination with immunosuppressants) modulate this injury? To this end, we introduce voxel-mirrored homotopic connectivity (VMHC), an innovative indicator based on resting-state functional MRI (rs-fMRI), which can reflect the changes in the isotropic connection between each voxel in one cerebral hemisphere and the mirror voxel in the contralateral cerebral hemisphere, and can reflect the information integration function between the cerebral hemispheres by analysing the synchronous activity of neurons between the cerebral hemispheres. The reliability and reproducibility of VMHC have been proven. Significant progress has been made in recent years and has been widely used to assess cerebral hemisphere integration in neurodegenerative and psychiatric disorders.^9 10^ Therefore, we will use VMHC to reveal microscale brain network imbalances that cannot be captured by traditional methods by quantifying the synchronicity of time series of bilaterally symmetric brain,^11^ providing insights for the visualisation of potential brain injury and the effects of drugs on patients with SLE without obvious neuropsychiatric manifestations.

## Methods

### Study participants and grouping

Patients with SLE treated in the Department of Rheumatology and Immunology of the First Affiliated Hospital of Kunming Medical University were recruited as the case group, and healthy volunteers of the same period were recruited as healthy controls (HCs) for the study. The research process of collecting clinical data and MRI imaging data was carried out using a standardised protocol and was followed by the same investigator throughout the study. Each study subject was interviewed by a rheumatologist and psychiatrist with extensive clinical experience, and a detailed and comprehensive physical examination was conducted, with a focus on neuropsychiatric lesions, and the inclusion and exclusion criteria were as follows.

#### Inclusion criteria for SLE group

(1) Patients with SLE who meet the SLE classification criteria formulated by the American College of Rheumatology (ACR) in 1997, have not been previously diagnosed with NPSLE, have not yet developed obvious neuropsychiatric symptoms and have normal routine brain MRI T1-weighted imaging (T1WI) and T2-weighted imaging (T2WI) scans; (2) aged 18–50 years; (3) right-handedness as assessed by the Edinburgh Handedness Scale; (4) those who voluntarily participate in this study know and sign the informed consent form and can cooperate with MRI examination and questionnaire evaluation.

#### Exclusion criteria for SLE group

(1) Patients with a history of severe head injury; (2) patients with a history of organic encephalopathy or head trauma that interferes with the structure of the brain, a history of craniocerebral surgery, Parkinson’s disease or epilepsy, cerebrovascular accident or visual impairment, cranial nerve disease, or severe persistent headache and other neurological diseases; (3) history of severe mental illness such as depression, anxiety and schizophrenia; (4) patients with a history of alcoholism and drug abuse or who have received antidepressant or antipsychotic medication; (5) patients with other autoimmune diseases; (6) patients with severe clinical manifestations that may affect brain structures, such as severe hypertension, diabetes mellitus or renal insufficiency; (7) there are contraindications to MRI scanning, such as those with claustrophobia or those with metal foreign bodies like dentures; (8) patients who are unable to cooperate with the completion of the scale assessment and questionnaire filling.

#### Inclusion criteria for the HC group

(1) Aged 18–50 years; (2) right-handedness as assessed by the Edinburgh Handedness Scale; (3) no serious physical or mental illness in the past, physical and mental health; (4) participants with normal T1WI and T2WI scans of conventional brain MRI; (5) those with a normal comprehensive physical examination performed by an experienced rheumatologist; neurological examination performed by an experienced psychiatrist and screening with normal results using the Structured Clinical Interview for Non-Patients from the Fourth Edition of the Diagnostic and Statistical Manual of Mental Disorders; (6) non-lactating or pregnant women; (7) participants voluntarily participated in this study and signed the informed consent form and were able to cooperate with MRI examination and questionnaire evaluation.

All subjects and/or their legal guardians have been informed in detail of the purpose of the study and all research contents before enrolment.

### Grouping policies

Patients with SLE were divided into three groups based on the 3-month treatment regimen prior to MRI scans:

*Treatment-naïve group*: not treated with GC, immunosuppressants or immunomodulators.

*GC monotherapy group*: continuous oral GC (such as prednisone and methylprednisolone for ≥3 months).

*Combination therapy group*: GC combined with CTX (oral or pulse venous therapy) and/or HCQ (200–400 mg/day for ≥3 months).

### Data collection and processing

(1) Demographic information: gender, age, education level, etc. (2) Clinically relevant indicators: course of disease, autoantibodies and other laboratory indicators; we retrospectively collected serum autoantibody profiles for all patients with SLE at the time of MRI scanning, which included: anti-double-stranded DNA (anti-dsDNA), anti-Smith, anti-U1 ribonucleoprotein, anti-Sjögren’s syndrome-related antigen A 52 kilodalton, anti-Sjögren’s syndrome-related antigen A 60 kilodalton, anti-Sjögren’s syndrome type B, anti-histones, anti-P0 and anti-nucleosome antibodies. The course of the disease is defined as the time from the initial clinical presentation that can be definitively attributed to SLE to the date of MRI acquisition; all clinical manifestations and laboratory tests were documented according to the criteria of ACR. (3) Drug use: data on the total dose of GC and immunosuppressants used between the start of medication and the day of MRI acquisition were obtained through patient interviews and detailed review of case data. Data were normalised by switching to prednisone equivalent doses of oral and parenteral corticosteroids. The cumulative dose is calculated by multiplying the sum of the daily doses by the number of days of treatment. The total dose of oral and intravenous GC is calculated by converting to an equivalent dose of prednisone. (4) SLE Disease Activity Index 2000 (SLEDAI-2K) was used to assess the disease activity of patients with SLE. (5) Mental scale assessment: Hamilton Anxiety Scale (HAMA), HAMD and MMSE were used to evaluate patients’ anxiety, depression and cognitive function, respectively. All scales were assessed by an experienced psychiatrist 2 days before or after the MRI examination. We do not rule out mood disorders and cognitive impairment in this study because these two symptoms are traditionally considered ‘non-severe’ and ‘functional’ disorders of the brain and are distinct from other obvious CNS disorders.

### Image data acquisition

#### Acquisition of magnetic resonance data

All image acquisition was performed by an experienced radiologist. All subjects completed data acquisition with a birdcage head coil on the same 1.5T MRI scanner (Twinspeed; GE Medical Systems), and the scan range covered the whole brain. Subjects in the supine position use foam support pads to reduce head movements, wear an eye mask during scanning, relax and keep the body still. MRI scan in blood pressure, pulse and breathing monitoring and scan chamber video monitoring prompts subjects at the end of each sequence to keep the subject awake. Routine T1WI and T2WI scans were performed to exclude obvious structural abnormalities with scan parameters as follows: T1WI uses a fluid-attenuated inversion recovery, axial view, repetition time (TR)=1800 ms, echo time (TE)=8.9 ms, inversion time (TI)=700 ms, number of excitations (NEX)=2.00, floor thickness=5 mm, interlamellar spacing=1 mm, flip angle (FA)=90°, field of view (FOV)=24 cm×24 cm, imaging matrix=256×256; T2WI: TR=12 000 ms, TE=88.4 ms, floor thickness=6 mm, interlamellar spacing=6 mm, FA=90°; rs-fMRI data using the 3D T1-weighted fast disturbance phase gradient echo sequence with the following parameters: TR=10.5 ms, TE=2.0 ms, TI=350 ms, floor thickness=1.8 mm, no layer interval, imaging matrix=256×256, FOV=24 cm×18 cm, FA=15°, spatial resolution=0.94 mm×0.94 mm×0.94 mm, number of plies=172; fMRI data: gradient echo sequence using echo planar imaging (EPI) technology with the following parameters: TR=2000 ms, TE=40 ms, NEX=2.0, imaging matrix=6464, FOV=24 cm, FA=90°, floor thickness=5 mm, interlamellar spacing=1 mm, number of plies=24, time point=160, a total of 320 s.

#### Imaging data processing

Data processing and analysis is based on the Matlab2018b platform and completed using a toolbox for Data Processing & Analysis for (Resting-State) Brain Imaging (DPABI) (http://rfmri.org/dpabi) statistical analysis software.^12^ Specific steps include: (1) Format conversion. (2) Remove the data of the first 10 time points, with the purpose of excluding the signal pollution caused by non-objective reasons, such as signal instability in the early stage and the need of time for the subjects to adapt to the scanning stage. (3) Slice timing was performed using the Fourier difference correction method using the middle layer as a reference. (4) Realign: correct for the small head displacement of the subject during the examination. (5) Quality control of functional images and structural images; grey matter, white matter and cerebrospinal fluid (CSF) were separated by New Segment+DARTEL. (6) Nuisance covariates regression: regression covariates, such as the interference signal and linear drift of white matter and CSF, head movement (Friston 24 parameter), etc. To minimise the influence of head motion on functional connectivity estimates, participants with max head motion translation >2.0 mm or rotation >2.0° were excluded. This threshold was selected based on prior rs-fMRI studies using similar motion criteria to ensure data quality while retaining a representative sample.^12^ (7) Gaussian smoothing check data with 6 mm full width at half maximum (FWHM) for spatial smoothing processing. (8) Filter: a temporal band-pass filter (0.01–0.1 Hz) was applied to retain low-frequency fluctuations intrinsic to the blood oxygen level dependent (BOLD) signal while minimising the effects of high-frequency physiological noise (eg, cardiac and respiratory cycles). This frequency range is widely adopted in rs-fMRI preprocessing pipelines.^13^ (9) Spatial normalisation and resampling: apply the functional images of each subject to the same template for subsequent comparison. This study used the EPI template based on the standard space of the Montreal Neurological Institute and resampled the voxels to sizes of 3 mm×3 mm×3 mm.

#### VMHC calculation

Extract the time series of each voxel in one side of the subject’s brain hemisphere, then calculate the Pearson correlation coefficient between each time series and the symmetric voxel of the mirror voxels in the contralateral hemisphere. Finally, the resulting correlation coefficients were converted to Z values by Fisher Z transformation to improve normality to generate whole-brain VMHC maps for each subject. For detailed calculation methods, see the report by Zuo *et al*.^14^

### Patient and public involvement

Patients or the public were not involved in the design, conduct, reporting or dissemination plans of our research.

### Statistical analysis

Statistical analyses of demographic data and clinical indicators were performed using the SPSS V.26.0 software. Χ^2^ test or Fisher’s exact test was used for counting data. Non-parametric Kolmogorov-Smirnov test is used to test the normality of the measurement data. Normally distributed data are represented by mean±SD (x̅±s), and non-normally distributed data by median (M) (Q1, Q3). To compare normally distributed measurement data between two groups using two independent sample t-tests and Mann-Whitney U test for non-normally distributed data, comparisons between multiple groups were performed by analysis of variance or Kruskal-Wallis H test with least significant difference (LSD) correction post hoc, with statistical significance set at p<0.05.

MRI data were analysed using the DPABI,^12^ and two independent sample t-tests were used to compare the differences of VMHC values between SLE group and HC group. The VMHC values of the different brain regions were extracted and correlated with SLEDAI, HAMA and HAMD by Spearman correlation analysis. Analysis of covariance (ANCOVA) was used to compare the differences of VMHC between the four groups, with sex, age and the framewise displacement parameter of head movement as covariates. Within the spatial mask of the four ANCOVA results, two independent sample t-tests were used to compare the differences between HC group, treatment-naïve group, GC group and GC combined with CTX and/or HCQ group. Gaussian random field (GRF) was used for multiple comparison correction (voxel p<0.005, cluster p<0.01). We also included the status of autoantibodies (positive and negative) as a binary covariate and readopted ANCOVA to conduct a sensitivity analysis on the differences in VMHC among the various groups. To avoid the complication of the model caused by too many covariates, as well as the risk of multicollinearity and the reduction in statistical power, we will only include autoantibodies with an intergroup difference of p<0.1. Use the DPABI attachment viewer to view and visualise the results.

Post hoc power calculation: After ANCOVA, we used G*Power 3.1 to compute the achieved power. The input consisted of the observed effect size (converted from partial η² to Cohen’s f), number of groups, number of covariates and the total sample size. Alpha was set at 0.05. This step verifies whether the current design could detect the VMHC differences.

## Results

### Analysis of demographic and clinical indicators and related psychiatric scales

A total of 234 subjects were recruited in a continuous group who met the inclusion and exclusion criteria, including 140 in the SLE group and 94 in the HC group. There was no significant difference in gender, age and years of education between the two groups (see table 1). In this study, missing data were primarily due to incomplete responses to the psychological scales (HAMD, HAMA, MMSE) or failure to complete all clinical assessments. Specifically, among the 140 recruited patients with SLE, 53 did not complete the psychological scale assessments (HAMD, HAMA, MMSE) due to time constraints or personal reasons. Additionally, eight patients had incomplete clinical laboratory data. We conducted a complete case analysis (available case analysis), excluding patients with missing data from the relevant analyses involving these scales. Based on the treatment before the MRI scan, 38 cases were excluded due to the use of leflunomide, methotrexate and other immunosuppressants. The screened patients with SLE were divided into treatment-naïve group (n=22), GC monotherapy group (n=30), GC combined with CTX and/or HCQ group (n=50) and HC group (n=29). There were no significant differences in gender, age and years of education among the four groups. No significant difference was observed in the cumulative GC amount between the GC monotherapy group and the GC combined with CTX and/or HCQ group. There were no significant differences in SLEDAI, HAMD and HAMA among the three SLE subgroups. Χ^2^ tests revealed that only anti-dsDNA antibody positivity differed significantly across the three treatment groups (p=0.048), while the remaining eight antibodies showed no significant intergroup differences (p>0.25) (see table 2). The combination therapy group was further subdivided into three regimens: GC+CTX (n=7), GC+HCQ (n=33) and GC+CTX+HCQ (n=10). Baseline characteristics across these subgroups were comparable, with no significant differences observed (online supplemental table 1).

**Table 1:** Demographic and clinical data results between HC and SLE groups

	HC (n=94)	SLE (n=140)	*Z*/Χ^2^	P value
Gender (male/female)	23/71	22/118	2.775	0.096
Age (years)	28 (25, 34)	29 (24, 35)	0.244	0.808
Years of education (year)	15 (12, 15)	14 (9, 16)	1.880	0.060
SLEDAI	–	10 (6, 16)	–	–
HAMD	0 (0, 0)	9 (3, 13) (n=87)	10.888	<0.001
HAMA	0 (0, 0)	6 (3, 11) (n=87)	10.860	<0.001
MMSE	30 (30, 30)	28 (25, 29) (n=87)	9.800	<0.001

Symbol ‘–’ denotes not applicable.

HAMA, Hamilton Anxiety Scale; HAMD, Hamilton Depression Scale; HC, healthy control; MMSE, Mini-Mental State Examination; SLEDAI, SLE Disease Activity Index.

**Table 2:** Demographic and clinical data of different drug therapy groups

	HC (n=29)	Treatment naïve (n=22)	GC monotherapy (n=30)	GC combined with CTX and/or HCQ (n=50)	*H*/*F*/Χ^2^/Fisher/*Z/t*	P value
Gender (male/female)	7/22	3/19	4/26	8/42	1.483	0.723
Age (years)	27 (25, 36)	29.5 (25.75, 36.5)	26.5 (24, 33.25)	27.5 (22.75, 34.25)	2.380	0.497
Years of education (year)	15 (9.5, 16)	10 (8, 14)	13 (9, 16)	14 (9, 16)	6.031	0.110
GC accumulation (g)	–	–	1.02 (0.25, 5.21) (n=29)	1.74 (0.6, 9.76) (n=42)	−1.755	0.079
CTX accumulation (g)	–	–	–	0 (0, 0) (n=47)		–
HCQ accumulation (g)	–	–	–	2.8 (4, 79.4) (n=43)		–
SLEDAI	–	11.5 (7, 19.25)	10.5 (5.75, 17)	8.5 (5, 15.25)	2.952	0.229*
HAMD	0 (0, 0)	10 (5, 16) (n=19)	10 (6.5, 13.5) (n=21)	7.5 (2, 11) (n=28)	4.461	0.107*
HAMA	0 (0, 0)	7 (4, 13) (n=19)	8 (3, 12) (n=21)	5 (2.5, 8.75) (n=28)	1.955	0.376*
MMSE	30 (30, 30)	27 (23, 30) (n=19)	28 (26, 29) (n=21)	28 (27.25, 29.75) (n=28)	2.653	0.265*
Anti-dsDNA antibody (%)		16/22 (72.72)	19/30 (63.33)	22/50 (44.00)	6.071	0.048†
Anti-Sm antibody (%)		11/22 (50.00)	15/30 (50.00)	23/50 (46.00)	0.163	0.922
Anti-U1RNP antibody (%)		8/22 (36.36)	8/30 (26.67)	18/50 (36.00)	0.851	0.653
Anti-SSA52KD antibody (%)		11/22 (50.00)	16/30 (53.33)	23/50 (46.00)	0.414	0.813
Anti-SSA60KD antibody (%)		13/22 (59.09)	16/30 (53.33)	30/50 (60.00)	0.360	0.835
Anti-SSB antibody (%)		10/22 (45.45)	9/30 (30.00)	13/50 (26.00)	2.723	0.256
Anti-histones antibody (%)		9/22 (40.91)	15/30 (50.00)	20/50 (40.00)	0.821	0.663
Anti-P0 antibody (%)		10/22 (45.45)	14/30 (46.67)	20/50 (40.00)	0.401	0.818
Anti-nucleosome antibody (%)		10/22 (45.45)	17/30 (56.67)	19/50 (38.00)	2.640	0.267

Symbol ‘–‘ denotes not applicable.

* Results of Kruskal-Wallis H test among treatment-naïve, GC monotherapy and GC combined with CTX and/or HCQ groups.

† P<0.05.

Anti-dsDNA antibody, anti-double-stranded DNA antibody; Anti-P0 antibody, anti-ribosomal P0 protein autoantibody; Anti-Sm antibody, anti-Smith antibody; Anti-SSA52KD antibody, anti-Sjögren’s syndrome-related antigen A 52 kilodalton antibody; Anti-SSA60KD antibody, anti-Sjögren’s syndrome-related antigen A 60 kilodalton antibody; Anti-SSB antibody, anti-Sjögren’s syndrome type B antibody; Anti-U1RNP antibody, anti-U1 ribonucleoprotein antibody; CTX, cyclophosphamide; GC, glucocorticoid; HAMA, Hamilton Anxiety Scale; HAMD, Hamilton Depression Scale; HC, healthy control; HCQ, hydroxychloroquine; MMSE, Mini-Mental State Examination; SLEDAI, SLE Disease Activity Index.

### Results of VMHC analysis between HC and SLE groups

**Figure 1:**
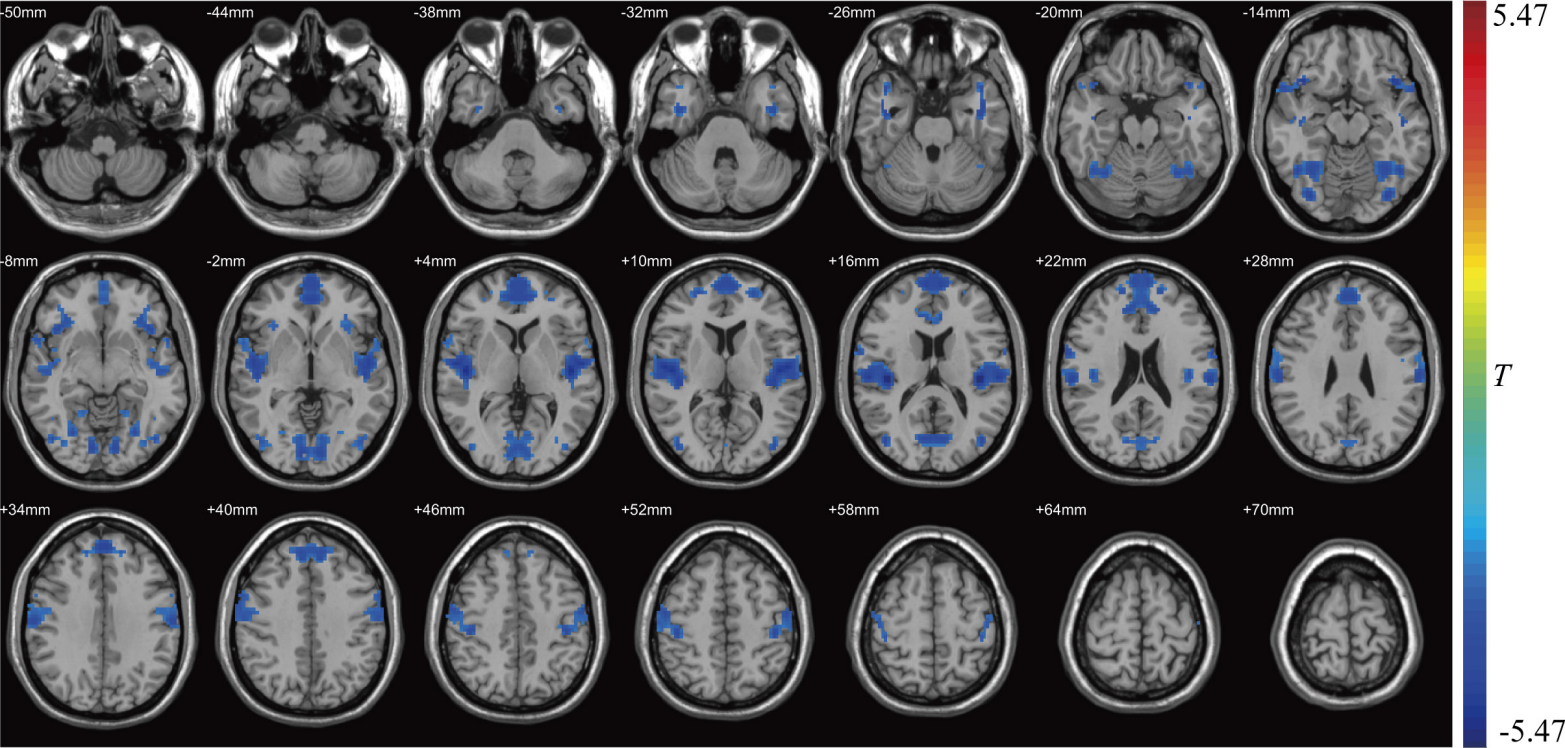
There were different VMHC clusters between the SLE group and the HC group. Red indicates that the VMHC in the SLE group was stronger than that in the HC group. Blue indicates that the VMHC in the SLE group was weaker than that in the HC group. The colour bar on the right side indicates the *t* value. HC, healthy control; VMHC, voxel-mirrored homotopic connectivity.

VMHC in superior temporal gyrus (*t*=−2.835), medial superior frontal gyrus (*t*=−2.837), calcarine fissure and surrounding cortex (*t*=−2.835) and middle occipital gyrus (*t*=−2.837) of SLE group was lower than that of HC group (see table 3 and figure 1). VMHC in bilateral superior temporal gyrus (r_s_=−0.250, p=0.024) and medial superior frontal gyrus (r_s_=−0.246, p=0.026) was negatively correlated with HAMD score. VMHC of medial superior frontal gyrus was negatively correlated with HAMA score (r_s_=−0.239, p=0.031). VMHC values in differential brain regions were not significantly correlated with SLEDAI.

**Table 3:** Clusters with significant VMHC differences between the SLE (n=140) group and HC group (n=94)

Cluster	Brain region	Voxel (n)	MNI spatial coordinates			*t*
			X	Y	Z	
**SLE group<HC group**						
Cluster 1	Superior temporal gyrus	1191	±45	3	−9	−2.835
Cluster 2	Medial superior frontal gyrus	974	±9	63	3	−2.837
Cluster 3	Cortex around the calcarine fissure	430	±12	−90	3	−2.835
Cluster 4	Middle occipital gyrus	305	±36	−81	6	−2.837

Results corrected by GRF: voxel p<0.005, cluster p<0.01.

GRF, Gaussian random field; HC, healthy control; MNI, Montreal Neurological Institute standard spatial template; VMHC, voxel-mirrored homotopic connectivity.

### Results of VMHC analysis among different drug treatment regimen SLE subgroups and HC group

**Figure 2:**
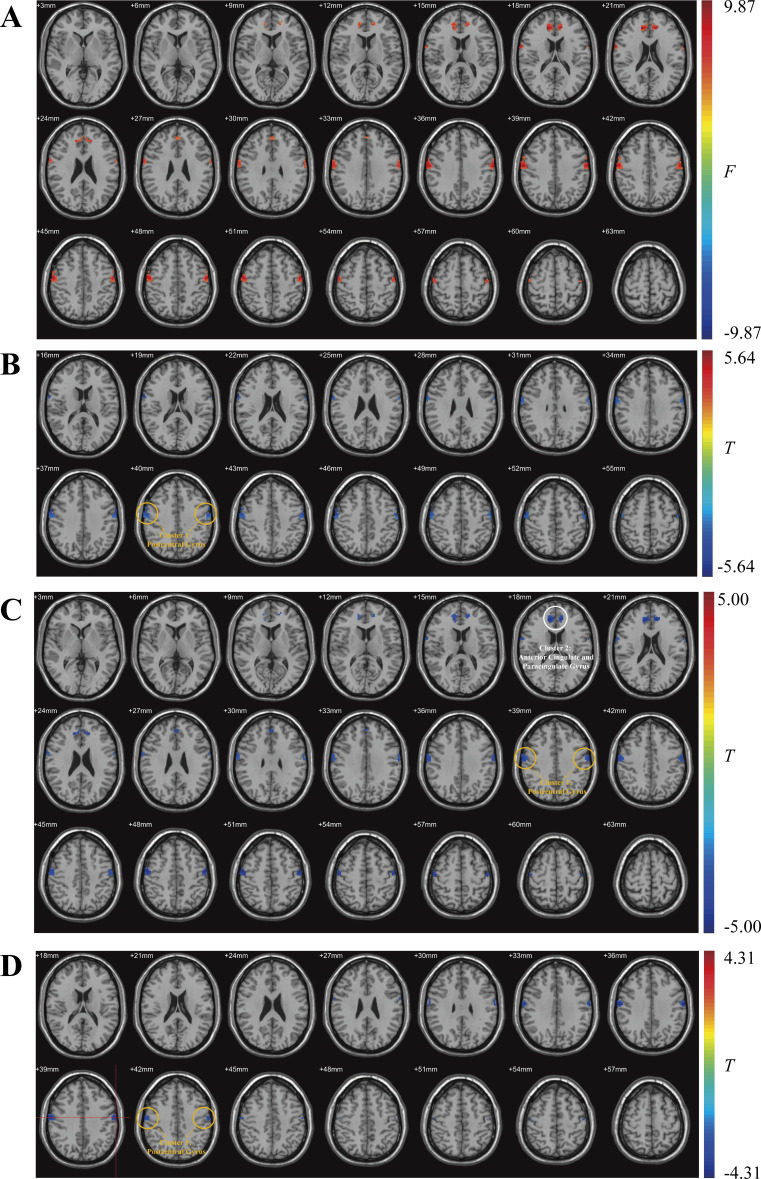
Brain regions with differences in VMHC were compared between groups. (A) ANCOVA analysis results of VMHC values in HC, treatment-naïve, GC monotherapy and GC combined with CTX and/or HCQ groups. (B) Brain regions with different VMHC between treatment-naïve and HC groups. Red indicates that the VMHC in the treatment-naïve group was higher than that in the HC group. Blue indicates that the VMHC in the treatment-naïve group was lower than that in the HC group. (C) Brain regions with different VMHC between GC group and HC group. Red indicates that VMHC in the GC group is higher than that in HC group. Blue indicates that VMHC in GC group is lower than that in HC group. (D) Brain regions with different VMHC between GC group and GC combined with CTX and/or HCQ group. Red indicates that VMHC in GC group is higher than that in GC combined with CTX and/or HCQ group. Blue indicates that VMHC in GC group is lower than that in GC combined with CTX and/or HCQ group. The colour bar indicates the *F*/*t* value. ANCOVA, analysis of covariance; CTX, cyclophosphamide; GC, glucocorticoid; HC, healthy control; HCQ, hydroxychloroquine; VMHC, voxel-mirrored homotopic connectivity.

The VMHC of HC group, treatment-naïve group, GC monotherapy group and GC combined with CTX and/or HCQ group was analysed by covariance analysis. After correction of LSD and GRF multiple comparisons (voxel p<0.005, cluster p<0.01), the VMHC of the two clusters was significantly different between the four groups (table 4, figure 2A).

**Table 4:** Correlation analysis of the mean VMHC of each abnormal brain region in the SLE group with clinical data and related psychological scales

	Brain region	r_s_	P value
HAMD	Superior temporal gyrus	−0.250	0.024
	Medial superior frontal gyrus	−0.246	0.026
HAMA	Medial superior frontal gyrus	−0.239	0.031

HAMA, Hamilton Anxiety Scale; HAMD, Hamilton Depression Scale; VMHC, voxel-mirrored homotopic connectivity.

Post hoc analysis showed that compared with the HC group, one cluster had a decreased VMHC value in the treatment-naïve group (table 5, figure 2B). The VMHC value of two clusters in the GC monotherapy group was lower than that of the HC group (table 5, figure 2C). Compared with the GC combined with CTX and/or HCQ group, VMHC in the GC monotherapy group was decreased (table 5, figure 2D). There was no significant difference in VMHC between the GC combined with CTX and/or HCQ group and the HC group. At the same time, no clusters with significant differences were found between the treatment-naïve group and the GC monotherapy group, as well as between the treatment-naïve group and the GC combined with CTX and/or HCQ group.

**Table 5:** Differences in VMHC between HC group, treatment-naïve group, GC group and GC with CTX and/or HCQ group

Cluster	Brain region	Voxel (n)	MNI spatial coordinates			*F/t*
			X	Y	Z	
ANCOVA of VMHC in HC, treatment-naïve, GC monotherapy and GC combined with CTX and/or HCQ groups						
Cluster 1	Postcentral/precentral gyrus	174	±54	−9	51	8.94232
Cluster 2	Anterior cingulate/paracingulate gyrus	121	±15	36	18	9.86819
Treatment-naïve group<HC group						
Cluster 1	Postcentral gyrus	113	±57	−15	39	−2.95347
GC monotherapy group<HC group						
Cluster 1	Postcentral gyrus	147	±57	−6	39	−2.99937
Cluster 2	Anterior cingulate/paracingulate gyrus	117	±12	36	9	−2.99937
GC monotherapy group<GC combined with CTX and/or HCQ group						
Cluster 1	Postcentral gyrus	60	±60	−6	39	−2.89338

Treatment naïve indicates unmedicated.

Results were corrected by GRF: voxel p<0.005, cluster p<0.01.

ANCOVA, analysis of covariance; CTX, cyclophosphamide; GC, glucocorticoid; GRF, Gaussian random field; HC, healthy control; HCQ, hydroxychloroquine; MNI, Montreal Neurological Institute standard spatial template; VMHC, voxel-mirrored homotopic connection.

### Effect of anti-dsDNA antibody status on VMHC

After adjusting for anti-dsDNA antibody status, the ANCOVA revealed no significant clusters under the original strict GRF threshold. However, under a more lenient but still acceptable threshold (voxel p<0.05, cluster p<0.01), significant clusters emerged in the postcentral/precentral gyrus and anterior cingulate cortex, spatially consistent with our primary findings (online supplemental table 2 and online supplemental figure 1). This suggests that while anti-dsDNA status may exert a minor modulatory effect, the primary treatment-related VMHC differences are independent of anti-dsDNA antibody distribution.

### Power estimation

For the two clusters showing significant group differences (table 5), partial η² was 0.213, corresponding to Cohen’s f=0.520 (large effect). With four groups, four covariates and the present n=131, G*Power yielded an observed power of 0.999. Even under a conservative balanced scenario (n=22 per group, total n=88), power remained at 0.987, indicating that the study was well powered to detect the observed VMHC effects.

## Discussion

SLE is a highly heterogeneous autoimmune disease, and its neuropsychiatric manifestations (NPSLE) are one of the core factors leading to disability and mortality in patients.^3^ Conventional wisdom has shown that brain injury in SLE is marked by overt neuropsychiatric symptoms (eg, epilepsy, psychotic seizures), and patients with non-NPSLE who lack these symptoms are often considered ‘normal brain function’. However, this perception ignores the potential chronic damage to the CNS from the autoimmune response. In this study, fMRI technology was used to reveal for the first time that patients with non-NPSLE had transhemispheric functional synergistic impairment and systematically elucidated the central role of GC combined with HCQ and/or CTX in repairing brain network function. This discovery reshapes the theoretical framework of SLE brain injury and provides quantifiable imaging biomarkers for early intervention, marking the paradigm shift of SLE management from ‘symptom-driven’ to ‘pathological prevention’.

### Imaging decoding and theoretical innovation of subclinical brain injury

The core findings of this study suggest that even in patients with non-NPSLE who lack typical neuropsychiatric symptoms, the VMHC of key brain regions, such as bilateral superior temporal gyrus (*t*=−2.835), medial superior frontal gyrus (*t*=−2.837), cortex around the calcarine fissure (*t*=−2.835) and the middle occipital gyrus (*t*=−2.837), has been significantly reduced. These brain regions are involved in language processing, emotion regulation and visual integration, and their disconnection may be used to explain the subclinical cognitive decline and mood swings that are common in patients with SLE.^15 16^

Notably, the reduction of VMHC was negatively correlated with the scores of the depression scale (HAMD, r_s_=−0.250, p=0.024) and the anxiety scale (HAMA, r_s_=−0.239, p=0.031), suggesting that functional brain network disorders may be the neural basis of affective symptoms. This finding overturns the traditional dualism of ‘no neuropsychiatric symptoms or no brain injury’, confirming that central involvement of SLE is a continuous pathological process that begins at a subclinical stage, rather than an isolated acute event. Although this study currently only observed a descriptive association between VMHC reduction and depression/anxiety scores, increasing neuroimmunological evidence suggests that peripheral and central interleukin 6 (IL-6) and tumour necrosis factor-alpha (TNF-α) and other inflammatory cytokines may be the key molecular bridges connecting this functional connectivity defect with emotional disorders. During the pathological process of SLE, the deposition of immune complexes can activate the complement system, attract inflammatory cells and release proinflammatory cytokines. These cytokines damage the blood-brain barrier (BBB) and trigger local inflammatory responses in brain regions, such as the limbic system, leading to imbalances in neurotransmitters and dysfunction of brain regions, ultimately manifesting as mental symptoms, such as depression and anxiety. The elevated IL-6 in CSF has been identified as a key biomarker of neuroinflammation in NPSLE, and its level is directly correlated with learning and memory deficits.^17^ Abe *et al*’s research found that stress activates microglia in the medial prefrontal cortex of MRL/lpr mice, leading to the upregulation of IL-12/23p40 protein and an increase in dendritic spines, thereby causing neuropsychiatric abnormalities.^18^ A small-sample study by Postal *et al* found that in patients with SLE with CNS involvement, the CSF levels of IL-12, interferon gamma (IFN-γ), TNF-α and IL-10 were elevated. Among them, IFN-γ was directly correlated with cerebral volume reduction, suggesting that it drives the immune mechanism causing global brain atrophy in SLE.^19^ Furthermore, longitudinal studies have confirmed that regardless of the presence of neurological or psychiatric symptoms, persistent high disease activity and elevated serum IFN-α independently promote axonal damage, grey matter/thalamus atrophy, expansion of white matter lesions and cognitive decline in patients with SLE, suggesting that strict control of disease activity and reduction of cytokine levels can improve brain outcomes.^20^

Based on this, we propose a continuum model of SLE brain injury: under the action of genetic predisposition and environmental triggers, peripheral autoantibodies (such as anti-ribosomal P protein antibodies, anti-NMDA receptor antibodies) penetrate into the centre through the damaged BBB, causing glial cell activation and abnormal synaptic plasticity, resulting in a gradual loss of the brain functional connectivity. As the disease progresses, this micronetwork imbalance accumulates to a threshold and translates into overt neuropsychiatric symptoms. For the first time, this model deconstructs the pathological process of SLE brain injury into a dynamic chain of dysfunction to symptom appearance, providing a unified framework for understanding disease heterogeneity. Future longitudinal studies that can confirm the synchronisation of cytokine decline and VMHC recovery after immunosuppressive treatment will push the current descriptive association towards a causal neuroimmune model.

### Neuroprotective mechanisms of combination therapy

At the therapeutic level, this study made a breakthrough discovery: although GC monotherapy can partially alleviate VMHC damage (higher than that in the untreated group), its effect has a significant ceiling and cannot restore brain network function to a healthy level. In contrast, GC combined with HCQ and/or CTX can completely reverse VMHC abnormalities and is significantly better than monotherapy. This suggests that the combination therapy may have a synergistic effect in improving brain network function, potentially through multitarget mechanisms involving HCQ and CTX.

From a mechanistic point of view, this synergy may be achieved through a threefold pathway: (1) Peripheral-central immune decoupling: HCQ reduces type I interferon production by inhibiting the Toll-like receptor 7/9 signalling pathway, thereby reducing the penetration of peripheral autoantibodies (eg, anti-dsDNA antibodies) into the centre,^21 22^ while CTX blocks the continuous production of autoantibodies by depleting the B cell pool.^23^ The two drugs act synergistically on different stages of antibody generation and migration, providing a theoretical basis for significantly reducing the titre of CSF autoantibodies. (2) BBB repair: the anti-inflammatory properties of HCQ can inhibit the activity of matrix metalloproteinase 9, thereby producing immunomodulatory effects by reducing the degradation of BBB tight junction proteins; HCQ also attenuates BBB damage and increased brain fluid content in mice by inhibiting the downregulation of Claudin-5 membrane efflux.^24^ HCQ has also been found to alleviate the damage to the BBB and brain oedema by reducing neuroinflammation, the activation and aggregation of microglia and the infiltration of immune cells in the brain, and to upregulate the expression of tight junctions.^25^ (3) Microglia cell regulation: HCQ can inhibit the STAT3 protein in microglia, neurons and astrocytes, and reduce the level of neuroinflammation^26^; CTX reduces the infiltration of inflammatory cells in the brain and lowers the levels of proinflammatory factors. This helps alleviate neuroinflammation and thereby protect the brain tissue from autoimmune attacks. Therefore, CTX is used as the first-line treatment for severe CNS inflammatory diseases that have failed, such as CNS vasculitis, optic neuritis and myelitis, autoimmune encephalitis and tumour-associated multiple sclerosis.^27 28^ This dual regulation remodels the central immune microenvironment and promotes the restoration of synaptic plasticity.

In this study, although the positive rate of anti-dsDNA antibodies varied significantly among different drug treatment groups, when it was included as a covariate in the analysis, it did not significantly affect the intergroup differences in VMHC. This finding suggests that anti-dsDNA antibodies are not the main driving factor for the impact of drug treatment on brain functional connectivity. The subgroups of anti-dsDNA antibodies (such as DNRAbs) can cross-react with N-methyl-D-aspartate receptors, and after crossing the BBB, they can trigger neuroinflammation, leading to cognitive impairment and abnormal brain imaging.^29 30^ But imaging studies have also indicated that the positive antibody status is not always consistent with brain structure or functional damage.^31^ Therefore, we speculate that anti-dsDNA antibodies may more reflect the systemic inflammatory load rather than directly mediating the disruption of brain functional connectivity. The improvement effect of immunosuppressive treatment on VMHC may be achieved through broader peripheral-cerebral immune regulatory mechanisms, such as inhibiting proinflammatory cytokines, restoring BBB integrity or reducing the central infiltration of other more neurotoxic autoantibodies. Future longitudinal studies should explore whether seroconversion or titre reduction following immunosuppressive therapy parallels restoration of VMHC integrity.

These findings confirm for the first time from the functional imaging level that the combination of immunomodulators can break through the therapeutic bottleneck of GC through multitarget synergy, providing direct evidence for optimising the treatment of SLE. In clinical practice, early application of combination regimens may be a key strategy to prevent the progression of NPSLE. We fully acknowledge that a longitudinal, within-subject design would provide the highest level of evidence for drug-induced brain function changes. Nevertheless, in the current cross-sectional study, we deliberately incorporated a treatment-naïve group (n=22) who had never received GC, CTX or HCQ. By ensuring that the treatment-naïve group and the two medicated groups were comparable in baseline demographics, disease activity (SLEDAI-2K) and mood scores (HAMA/HAMD, table 2), we created a ‘baseline’ against which medication effects could be estimated. Under this design, the progressive recovery of VMHC (treatment naïve<HC, GC monotherapy<GC+CTX/HCQ≈HC) is best explained by treatment rather than spontaneous fluctuation, because untreated patients exhibited the lowest VMHC values while combination therapy restored VMHC to the HC level (figure 2). Although this approach cannot replace a genuine pre-post comparison, it offers the strongest inference feasible in the absence of longitudinal data and has been widely adopted in neuroimaging studies where ethical considerations preclude delaying necessary therapy. A prospective, within-subject trial is now underway to corroborate these findings.

### Advancing SLE assessment: from behavioural scales to brain network markers

Previous SLE studies have relied on neuropsychological scales (eg, MMSE, HAMD) to assess brain function, but these tools are highly susceptible to subjective factors and cannot capture subclinical impairment. In this study, however, three major advancements were achieved through the application of VMHC technology. First, VMHC demonstrated greater sensitivity than traditional methods for detecting subclinical injury, making it a valuable tool for early diagnosis. Second, VMHC allowed for the quantification of treatment response: while GC monotherapy partially alleviated VMHC damage, complete recovery of VMHC was observed in the combination treatment group, suggesting that VMHC can serve as an individualised predictor of treatment efficacy. Third, mechanism association analysis revealed that VMHC in the bilateral superior temporal gyrus, medial superior frontal gyrus and middle occipital gyrus was correlated with the functional connectivity strength of the default mode network (DMN), indicating that VMHC may be used as a surrogate marker of DMN integration ability. These advantages enable VMHC to transcend the limitations of traditional assessment tools and push SLE brain function assessment to a new dimension of objective quantification and circuit analysis. In clinical decision-making, patients with abnormal VMHC, even if asymptomatic, may benefit from early intensive therapy to avoid irreversible brain damage.

Despite important advances, there are limitations to this study that require careful interpretation. First, the cross-sectional design makes it difficult to establish the causal relationship between drug treatment and VMHC recovery; however, the inclusion of a treatment-naïve arm provides a proxy baseline that supports (but does not definitively prove) a medication effect. The intervention timeliness needs to be verified by longitudinal cohorts, such as paired scans before and after treatment. Second, the absence of biologics (eg, belimumab) or other immunosuppressants (eg, mycophenolate mofetil) may underestimate the potential benefits of combination therapy. Finally, the molecular mechanisms of VMHC abnormalities, such as specific autoantibodies and epigenetic regulation, still need to be analysed in combination with CSF proteomics and single-cell sequencing.

## Conclusion

Patients with SLE have a decline in mirror functional connectivity (FC) in multiple brain regions before obvious neuropsychiatric manifestations and may be associated with mood disorders of depression and anxiety. GC combined with CTX and/or HCQ can have better therapeutic effects in improving the FC of the brain mirror image than GC monotherapy.

## Supplementary material

10.1136/lupus-2025-001771 online supplemental file 1

10.1136/lupus-2025-001771 online supplemental figure 1

## Data Availability

Data are available upon reasonable request.
