# Supplementary material for: Role of combination immunotherapy in restoring brain synergistic functional connectivity in patients with systemic lupus erythematosus without overt neuropsychiatric manifestations
Source: Lupus Sci Med. 2025 Oct 29;12(2):e001771. doi: 10.1136/lupus-2025-001771 (PMC12574354; doi:10.1136/lupus-2025-001771)
Supplement: online supplemental file 1 [file lupus-12-2-s001.docx]

**Suppl Table 1.** Demographic and clinical data of GC combined with the CTX and / or HCQ groups

|  | GC+CTX  (n=7) | GC+HCQ  (n=33) | GC+CTX+HCQ  (n=10) | *H*/ Fisher | *P* |
| --- | --- | --- | --- | --- | --- |
| Proportion (%) | 14% | 66% | 20% | *-* | *-* |
| Gender (male/female) | 1/6 | 4/29 | 3/7 | 2.002 | 0.379 |
| Age (years) | 34（24,38） | 27（22,33） | 26（22.75,34） | 1.892 | 0.388 |
| Years of education (year) | 14（9,16） | 10（14,16） | 16（8.75,16） | 0.422 | 0.810 |
| SLEDAI | 8（6,16） | 8（5,13） | 13.5（3.75,20.25） | 1.724 | 0.422 |
| HAMD | 10 (5,14) | 6（1.5,11）（n=17） | 5（1.5,11）  （n=5） | 2.343 | 0.319 |
| HAMA | 7 (4,13) | 5（3,9）（n=17） | 4（2,7）（n=5） | 2.598 | 0.273 |
| MMSE | 27 (27,28) | 29（28,30）（n=17） | 28（28,29.5）（n=5） | 2.653 | 0.305 |

Note: HCs, healthy control; GC, corticosteroids; CTX, cyclophosphamide; HCQ, Hydroxychloroquine; SLEDAI, systemic lupus erythematosus disease activity index; MMSE, Mini-Mental State Examination; HAMA, Hamilton Anxiety Scale; HAMD, Hamilton Depression Scale; -, not applicable.

**Suppl Table 2.** VMHC differences across groups after adjusting for anti-dsDNA antibody status

| cluster | brain region | voxel number | MNI spatial coordinates | | | *F* |
| --- | --- | --- | --- | --- | --- | --- |
|  |  |  | X | Y | Z |  |
| Cluster 1 | postcentral gyrus and precentral gyrus | 380 | ±66 | -12 | 39 | 6.1520 |
| Cluster 1 | anterior cingulate | 340 | ±15 | 36 | 18 | 8.1129 |

Note: HCs, healthy control; Treatments-Naïve, unmedicated; GC, corticosteroids; CTX, cyclophosphamide; HCQ, hydroxychloroquine; VMHC, voxel-based mirror homotopic connection; MNI, Montreal Neurological Institute standard spatial template; results were corrected by GRF: Voxel *P* value <0.05, Cluster *P* value <0.01

**
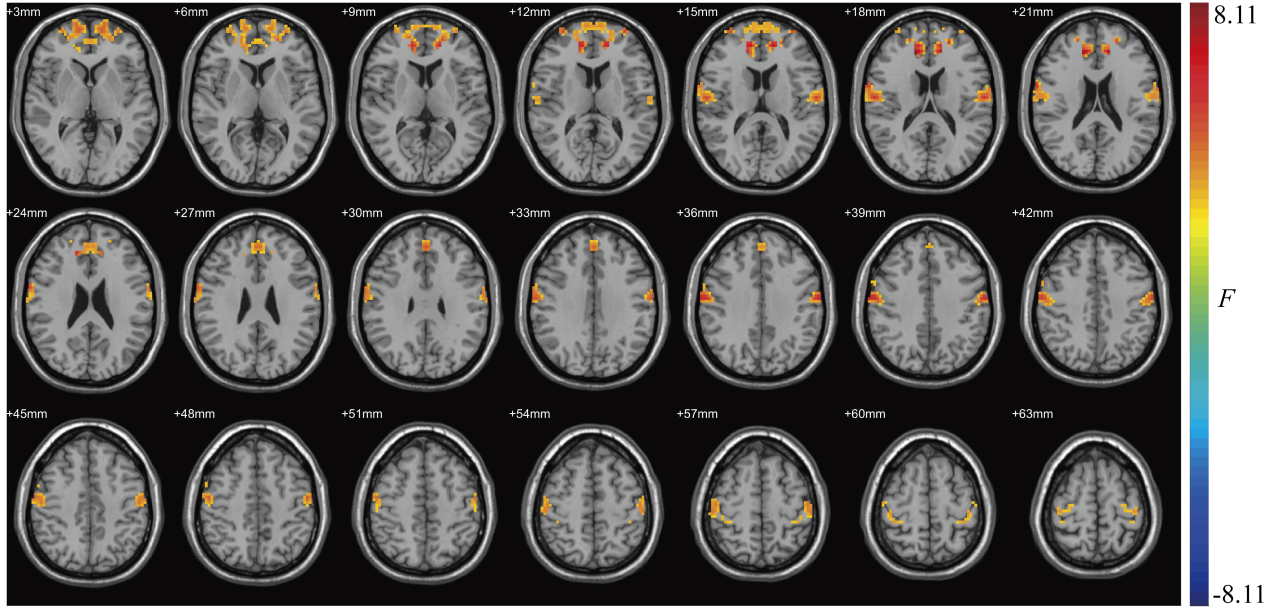
**

**Suppl Figure 1.** VMHC differences across groups after adjusting for anti-dsDNA antibody status

ANCOVA analysis results of VMHC values in HCs group, Treatments-Naïve, GC, GC combined with CTX and/or HCQ group; The color bar indicates the *F* value, results were corrected by GRF: Voxel *P* value <0.05, Cluster *P* value <0.01.
