# Supplementary figures and images for: Role of combination immunotherapy in restoring brain synergistic functional connectivity in patients with systemic lupus erythematosus without overt neuropsychiatric manifestations
Source: Lupus Sci Med. 2025 Oct 29;12(2):e001771. doi: 10.1136/lupus-2025-001771 (PMC12574354; doi:10.1136/lupus-2025-001771)

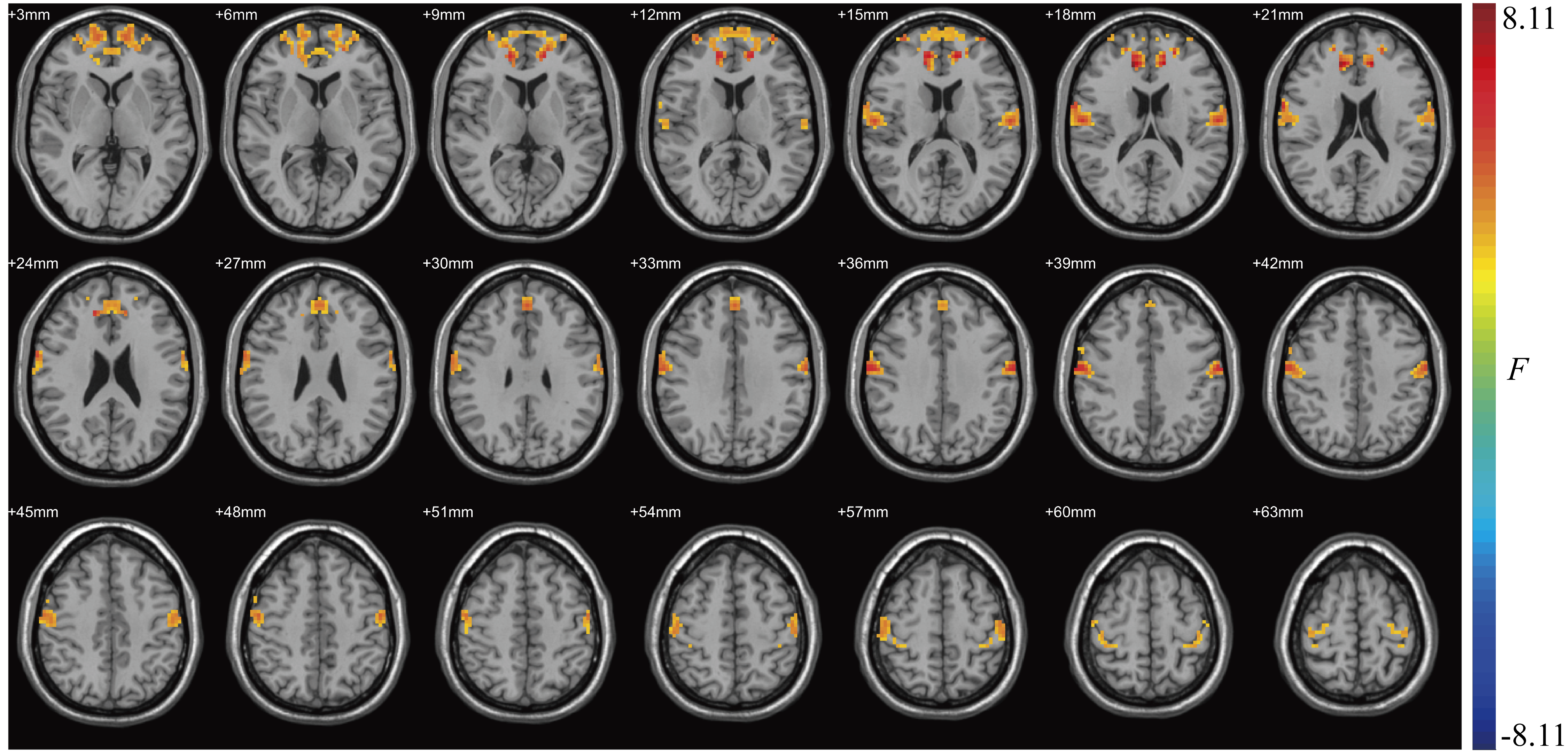

Supplement: online supplemental figure 1 [file lupus-12-2-s002.tif]
